# Dietary Docosahexaenoic Acid-Rich Supplementation Decreases Neurotoxic Lipid Mediators in Participants with Type 2 Diabetes and Neuropathic Pain

**DOI:** 10.3390/nu16234025

**Published:** 2024-11-24

**Authors:** Alfonso M. Durán, Francis Zamora, Marino De León

**Affiliations:** Center for Health Disparities and Molecular Medicine, Department of Basic Sciences, Loma Linda University School of Medicine, Loma Linda, CA 92350, USA; aduran@llu.edu (A.M.D.); fczamora@students.llu.edu (F.Z.)

**Keywords:** docosahexaenoic acid, lipidomics, autophagy, painful diabetic neuropathy

## Abstract

Background/Objectives: There is increasing evidence linking circulating neurotoxic lipids to the progression of chronic neuroinflammatory diseases in the peripheral and central nervous systems. Strategies to modify lipid profiles, such as docosahexaenoic acid (DHA)-rich supplementation, may aid in managing conditions like painful diabetic neuropathy (pDN). In a previous study, we demonstrated that three months of DHA supplementation significantly altered the metabolomic profile of patients with painful diabetic neuropathy (pDN), resulting in symptom improvement. This study investigates whether DHA-rich supplementation reduces neurotoxic lipid mediators associated with pDN in individuals with type 2 diabetes mellitus (T2DM). Methods: Forty individuals with type 2 diabetes participated in the “En Balance-PLUS” study, attending weekly lifestyle and nutrition education sessions while receiving daily supplementation of 1000 mg DHA and 200 mg EPA. Pain levels were assessed using the Short-Form McGill Pain Questionnaire (SF-MPQ) at baseline and after three months. Blood serum samples collected at these time points underwent untargeted lipidomic analyses, with ELISA used to evaluate biomarkers of necrosis (MLKL), autophagy (ATG5), and lipid chaperone protein (FABP5). Results: Untargeted lipidomic analysis revealed that several neurotoxic-associated lipids significantly decreased after DHA-rich supplementation. Also, circulating levels of MLKL were reduced, while protein levels of ATG5 and FABP5 significantly increased. Conclusions: The reduction of circulating neurotoxic lipids and increase in neuroprotective lipids following DHA-rich supplementation are consistent with the reported roles of omega-3 polyunsaturated fatty acids (PUFAs) in reducing adverse symptoms associated with neuroinflammatory diseases and painful neuropathy.

## 1. Introduction

The treatment of painful diabetic neuropathy (pDN) remains inadequate, and it is considered a significant co-morbidity of this condition [1]. Pharmacological treatment only assists about a third of patients to achieve 50% pain relief, often complicated by side effects [2,3,4]. Until recently, there was a consensus that hyperglycemia was the primary driver of diabetic neuropathy (DN). However, growing evidence suggests that lipid substrate overload, commonly observed in T2DM, has been proposed as a significant contributing pathogenic factor to DN [1,5,6,7]. The need for novel targets in the treatment of DN stems from the 2012 Cochrane review, which indicated that intensive glycemic control only marginally improved neuropathy in multiple type 2 diabetes cohorts [8]. Furthermore, intensive glycemic control significantly increases the risk of severe hypoglycemic episodes and is associated with increased mortality in T2DM [9]. Since the publication of these findings, a directed focus on lipid mediators in the development of DN is intensifying [10,11,12].

Dorsal root ganglion (DRG) neurons and Schwann cells are the primary cells for intact sensory processing. DRG neurons depend on mitochondrial ATP production throughout the axon and rely on mitochondrial transport mechanisms to distribute mitochondria for normal nerve function [13]. When exposing DRG neurons to elevated levels of C16:0 and C18:0 saturated fatty acids (SFAs), a marked decrease in mitochondrial trafficking is observed [14,15]. Ultimately, increased levels of SFA lead to mitochondrial depolarization and impaired mitochondrial bioenergetics, triggering DRG apoptosis [12,13]. In line with these findings, the neuronal model of nerve growth factor-differentiated pheochromocytoma cells (NGF-PC12) exposed to elevated palmitic acid levels demonstrated significant alterations in mitochondrial transmembrane potential alongside increased mRNA expression of key genes regulating cell death and survival [16,17]. Notably, our research showed that treating neuronal cells under lipotoxic stress with DHA reduced necroptosis activity, as evidenced by a marked decrease in mixed lineage kinase domain-like protein (MLKL) levels, while simultaneously enhancing autophagy activity. Additionally, SFA substrate overload decreased β-oxidation, increasing available palmitoyl-CoA [18]. The increased availability of palmitoyl-CoA, in turn, raised the levels of ceramides to toxic levels, inhibiting axonal growth [19,20]. These studies indicate that an overabundance of certain SFAs impairs mitochondrial function and plays a critical role in peripheral neuropathy progression.

Omega-3 PUFAs have the potential to shield neuronal tissues from SFA-induced metabolic stress, as demonstrated in previous studies [16,21]. DHA, a well-known omega-3 PUFA, has been shown to protect and reverse palmitic acid-induced lipotoxicity in neuronal cell models by inhibiting mitochondrial membrane depolarization, a significant biochemical feature in the development of DN [16,19]. Furthermore, omega-3 PUFAs have been found to reduce nerve damage and alleviate neuropathic pain in various pain models [22,23,24,25]. Significantly, DHA effectively reduced ceramide production and associated oxidative stress [26]. These findings underscore the role of omega-3 PUFAs in counteracting key metabolic pathways associated with DN, suggesting their potential as a therapeutic intervention.

We demonstrated that the neuroprotective effects of DHA are mediated through its interaction with FABP5, a lipid chaperone protein integral to neuronal survival and repair mechanisms [27]. FABP5 regulates the balance of bioactive lipids, mitigates oxidative stress, and enhances cellular repair following injury [28,29,30]. Specifically, it exerts antioxidant effects by reducing reactive oxygen species (ROS), which aids in nerve regeneration and functional recovery. Moreover, FABP5 exhibits ligand-dependent neuroprotective functions; for example, its interaction with DHA enhances endocannabinoid signaling and attenuates pro-inflammatory pathways, thereby reducing nociceptive sensitization and contributing to analgesic effects [27]. These findings underscore the synergistic relationship between FABP5 and DHA, highlighting a potential therapeutic mechanism for addressing neuropathic pain and neuroinflammatory conditions.

Recognizing lipid metabolism’s pivotal role in DN’s pathogenesis, our study takes a unique approach. We aim to identify changes in associated neurotoxic lipids in diabetic patients who report neuropathic pain symptoms before and after dietary omega-3 PUFA supplementation. Furthermore, we sought to investigate proteins associated with DHA-induced neuroprotection (MLKL, ATG5, and FABP-5) in our human population. To our knowledge, no previous human study has investigated the global lipidomic profiles of T2DM patients reporting neuropathic pain or the effects of dietary omega-3 PUFA supplementation on these profiles. Our approach employs a quantitative untargeted lipidomic analysis, which allows for an unbiased interpretation of the lipid mediators involved in DN and the impact of dietary omega-3 PUFA supplementation on the resolution of these neurotoxic lipids.

## 2. Materials and Methods

### 2.1. Study Design and Population

The En Balance-Plus study, a longitudinal single-arm study, was conducted in Loma Linda, California, to assess the effects of nutrition and diabetes education on type 2 diabetes in a Latino population, primarily Mexican Americans. This study was conducted in accordance with the Declaration of Helsinki, and the protocol was approved by the Loma Linda University Institutional Review Board (LLUIRB# 5110318) on 14 November 2019 [31,32]; informed consent was obtained from all participants prior to their involvement in this study.

### 2.2. Intervention

The En Balance-Plus study evaluated the effects of DHA-enriched dietary supplementation on participants’ lipidomic profiles. Over a three-month period, participants received daily capsules containing 1000 mg of DHA and 200 mg of eicosapentaenoic acid (EPA) while attending weekly Spanish-language diabetes education sessions.

### 2.3. Data and Study Variables

We previously described methods in detail [31,32]. Briefly, samples were collected for all participants at baseline and three months post-intervention, including fasting blood plasma and serum samples. Serum samples were sent to Metabolon for complex lipidomic analysis. Complex lipids were extracted from serum samples using a modified version of Lofgren’s procedure [33].

Participants’ diets, medication use, and exercise habits were closely monitored throughout the three-month intervention to ensure that any changes identified in the untargeted lipidomic analysis could be attributed to the dietary supplementation. A paired statistical analysis method was utilized, comparing each participant’s post-intervention values to their baseline measurements [32], effectively minimizing the impact of external variables on the results.

### 2.4. Measurement of Neuropathic Pain Symptoms

Neuropathic pain symptoms were monitored as previously described [32]. At baseline, 26 participants reported neuropathic pain symptoms, with 14 classified as having low pain (sensory score < 7) and 12 as having moderate-to-high pain (sensory score > 7).

### 2.5. Measurements of MLKL, ATG5, and EFABP5 Serum Levels by ELISA

Fasting serum samples of all participants were collected at baseline and three months post-intervention. Specifically, mixed lineage kinase domain-like (MLKL), autophagy protein 5 (ATG5), and fatty acid-binding protein 5 (FABP5) serum levels were measured using a commercially available enzyme immunoassay according to manufacturers’ instructions (MBS9300811, MBS2602759, and MBS2613697, MyBioSource Inc., San Diego, CA).

### 2.6. Statistical Analysis

An occupational threshold of 70% was applied to lipidomic data, requiring complex lipids present in at least 70% of the participants to be considered for analysis. Statistical analyses were performed using Prism 9 (GraphPad Software, San Diego, CA, USA) and MetaboAnalyst 6.0 (accessed on 19 April 2024) [27]. A paired-sample *t*-test was used to analyze normally distributed continuous variables, while non-normally distributed continuous variables were evaluated using Wilcoxon signed-rank tests to determine significant differences between baseline and three months post-supplementation. Normality was assessed using the Kolmogorov–Smirnov and Shapiro–Wilk tests, and outliers were identified using Grubbs’s test via GraphPad (www.graphpad.com, accessed on 19 April 2024). To control for false discovery rates, *p*-values were adjusted and reported as q-values. Data are presented as mean ± SD, with statistical significance set at α = 0.05 unless otherwise noted.

## 3. Results

### 3.1. Lipidomic Data Analysis

**Figure 1:**
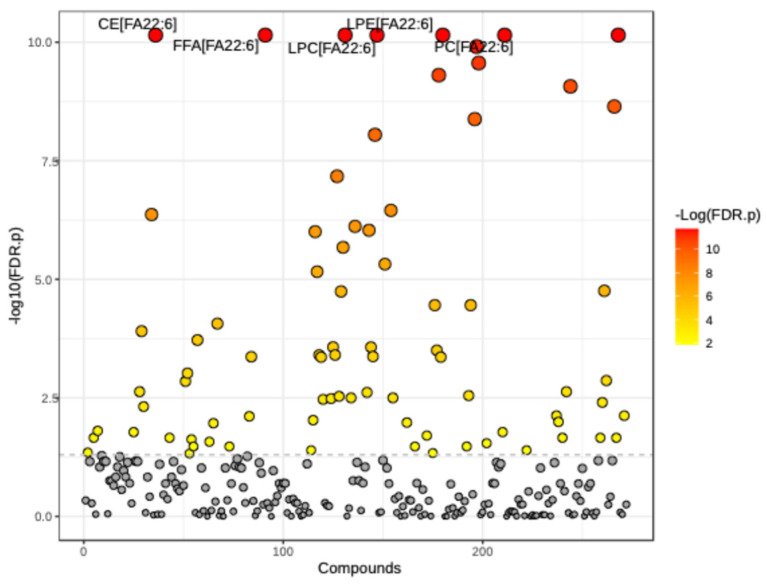
Matched pairs *t*-test with adjusted *p*-value (FDR) cutoff of 0.05. Eighty-one (81) lipid features were significantly different (*p* < 0.05) at three months.

We used Metabolon TrueMass^®^ complex lipid panel tests for several lipid species, concentrations, and compositions. For our analysis, we focused on fatty acid concentration [FA]. The panel consists of 283 known fatty acids. Of those, only 81 transformed [FA] significantly changed (*p* < 0.05) at three months on the matched pairs *t*-test (Figure 1). Specifically, the levels of 22 transformed [FA] were significantly increased (*p* < 0.05), while 59 were significantly decreased (*p* < 0.05) (Table 1). A summary of all significant [FA] is listed in Table 2. Interestingly, several known SFAs, MAG 16:0 and 18:0, LPC 16:0 and 18:0, LPE 18:0, and CER 16:0, considered to be neurotoxic and involved during neuropathy pathogenesis [10,34,35], significantly (*p* < 0.01) decreased post-DHA-rich dietary supplementation.

**Figure 2:**
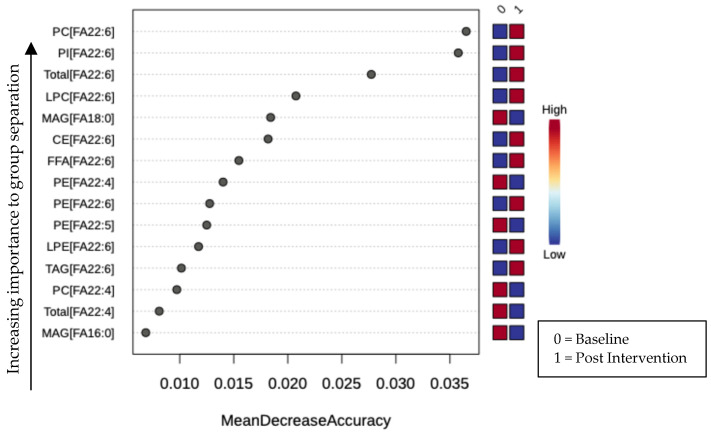
RF classification of serum samples collected at baseline (0) and three (3) months after DHA-rich supplementation (1). Classification was ~96% accurate for samples when a value of 50% would be expected by random chance. The plot for lipid features is important because it shows the top factors contributing to group separation.

Next, to assess the significance of fatty acid (FA) features associated with DHA-rich supplementation, we conducted random forest (RF) classification using fatty acid concentrations to predict sample classification at baseline or post-intervention. The classification of serum samples before and after DHA-rich supplementation achieved approximately 96% accuracy, significantly higher than the 50% accuracy expected by random chance. Fatty acid importance in group classification was quantified as the mean decrease in accuracy, as shown in Figure 2. The top 15 fatty acids contributing to group differentiation were predominantly enriched with DHA lipid derivatives. Notably, palmitic and stearic acid derivatives, MAG [FA16:0] and MAG [FA18:0], were among the most important features for group separation.

### 3.2. Assessment of Associated Markers of Painful Diabetic Neuropathy Levels by ELISA

#### 3.2.1. Measurement of MLKL Serum Levels by ELISA

**Figure 3:**
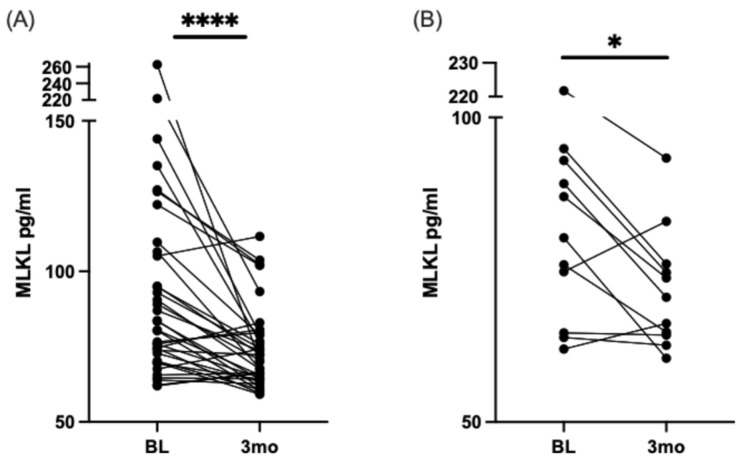
Mixed lineage kinase domain-like (MLKL) decreases after three (3) months of DHA-rich supplementation in participants with type 2 diabetes and neuropathic pain. Match pairs Wilcoxon test: (**A**) ELISA measured all participants’ serum levels of MLKL at baseline and three months. (**B**) Participants with moderately high pain at baseline and serum levels of MLKL, measured by ELISA, at baseline and three months. * *p* < 0.05, **** *p* < 0.0001.

In a previous study, our group demonstrated that SFA overload reduces the viability of fully differentiated pheochromocytoma clone 12 (PC12) cells by necrosis, an established in vitro model for studying the cellular mechanisms associated with DN [36,37]. However, we rescued the cells in the presence of SFA overload by co-treating the cells with DHA [16,17,38]. To determine if this mechanism holds in humans, we measured the serum levels of MLKL before and after DHA-rich supplementation in a human cohort. Figure 3 shows a significant decrease in MLKL serum levels post-DHA-rich supplementation in the overall group (regardless of pain status) and in the moderate-to-high pain group, as defined previously [31].

#### 3.2.2. Measurement of ATG5 Serum Levels by ELISA

**Figure 4:**
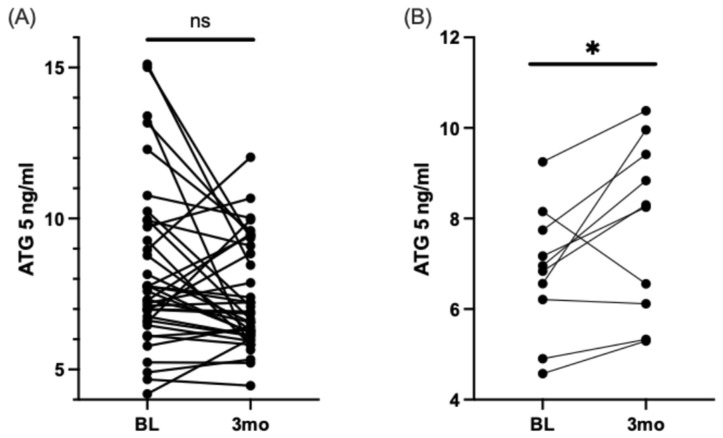
ATG5 increases after three months of DHA-rich supplementation in participants with type 2 diabetes and with painful DN. Match pairs Wilcoxon test: (**A**) All participants’ serum levels of ATG5, measured by ELISA, at baseline and three months. (**B**) Participants with moderately high pain at baseline and serum levels of ATG5, measured by ELISA, at baseline and three months. * *p* < 0.05.

Growing evidence suggests that critical pathways for cell maintenance, such as autophagy, play a crucial role in preventing and reversing neurodegenerative diseases, including DN [39,40,41,42]. Our previous research has shown that an overload of SFA can lead to neuronal cell toxicity, which can be alleviated by promoting autophagy [17]. For instance, we found that DHA can activate autophagy in the presence of SFA overload, and if autophagy is chemically inhibited, DHA’s ability to protect against SFA toxicity is diminished. Based on our previous findings linking lipids, autophagy, and DN, we measured the serum levels of ATG5 before and after DHA-rich supplementation in a human cohort. Interestingly, we discovered that ATG5 levels only increased in the moderate-to-high pain group following DHA-rich supplementation, as depicted in Figure 4.

#### 3.2.3. Measurement of FABP5 Serum Levels by ELISA

**Figure 5:**
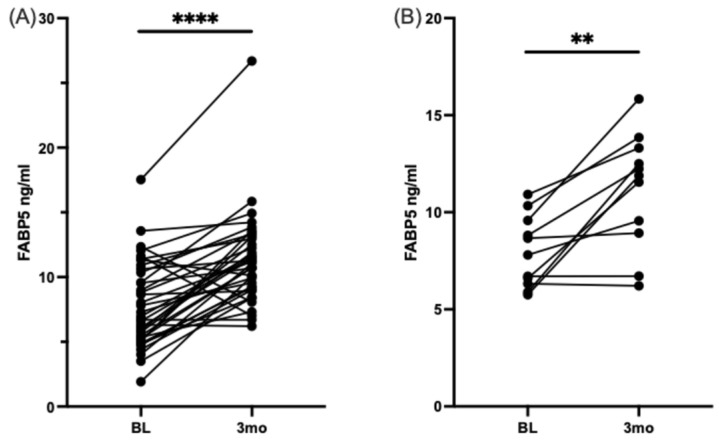
FABP5 increases after three months of DHA-rich supplementation in participants with type 2 diabetes and neuropathic pain. Match pairs Wilcoxon test: (**A**) ELISA measured all participants’ serum levels of FABP5 at baseline and three months. (**B**) Participants with moderately high pain at baseline and serum levels of FABP5, measured by ELISA, at baseline and three months. ** *p* < 0.01, **** *p* < 0.0001.

Our research group has shown that FABP5 plays an important role in neuronal development and neuronal injury and has antioxidant capacity [29,30,43,44]. We have also found that FABP5 is involved in locomotor recovery after spinal cord injury [27]. Additionally, FABP5 is essential for delivering neuroprotective fatty acids to the central nervous system, and a lack of FABP5 contributes to neuroinflammation [45,46]. Based on our experience with FABP5 and its neuroprotective effects in connection with DHA, we measured serum levels of FABP5 before and after DHA-rich supplementation in our cohort. As anticipated, we observed a significant increase in FABP5 serum levels after DHA-rich supplementation in the overall group (regardless of pain status) and the moderate-to-high pain group, as shown in Figure 5.

## 4. Discussion

### 4.1. Lipidomic Implications of DHA-Rich Supplementation in pDN

In addition to hyperglycemia, dyslipidemia is increasingly recognized as a significant contributing factor to neuropathy, especially in type 2 diabetes [1]. Moreover, many studies have demonstrated the harmful effects of SFA substrate overload on the nervous system [16,21,47,48]. Specifically, cellular studies showed that high concentrations of palmitic acid impaired sensory neuron mitochondrial trafficking and altered mitochondrial energy production [14]. Similarly, SFA overload in Schwann cells results in robust ER stress response, mitochondrial dysfunction, and increased oxidative stress [49], supporting the importance of dysregulated lipid metabolism in the pathogenesis of DN.

Specific palmitic and stearic acid derivatives are associated with the development of neuropathic pain. Long chain saturated fatty acids (LCSFAs) containing LPC and LPE, when in excess, increase neuronal reactive oxygen species in the dorsal root ganglion, causing altered neuronal activity and signaling [34,35]. LCSFAs containing LPC and LPE were also identified as pain biomarkers [50]. Our study showed that participants with T2DM who experienced pDN had decreased levels of C16:0 neurotoxic derivatives containing MAG (C16:0, C18:0), LPC (C16:0, C18:0), and LPE (C18:0) after three months of dietary supplementation. Particularly noteworthy was the observation that the overall level of serum C16:0 did not significantly change, indicating that the supplementation had a targeted effect on C16:0-associated metabolites. Furthermore, the fact that the serum C16:0 remained at baseline levels aligns with the participants’ report of not changing their diet aside from taking the provided DHA-rich supplementation. Lastly, our study is the first to report a significant decrease in associated neurotoxic LCSFA containing LPC and LPE after dietary omega-3 supplementation in patients with T2DM who reported neuropathic pain symptoms.

On the other hand, DHA-containing PC, LPE, and LPC have been reported to have neuroprotective properties [51]. Using RF analysis, our study demonstrates that DHA-PC is the most important complex lipid for distinguishing between baseline and post-intervention groups, indicating that DHA-PC levels could be a potential biomarker for pDN. Thus, the data suggest that individuals with T2DM and low levels of DHA-PC may be at a higher risk of developing pDN than those with higher levels of serum DHA-PC. Furthermore, we observed a significant increase in DHA-containing PC, LPC, and LPE serum levels after DHA-rich supplementation. Interestingly, DHA-containing PCs inhibit IL-6 signaling [51], an essential pro-inflammatory molecule involved in the abnormal activation of the NF-κB loop in DN [19].

Overall, we showed that complex lipid analysis identified key LCSFA derivatives of C16:0 and C18:0 involved in the pathogenesis of DN. More importantly, these findings indicate that dietary supplementation rich in DHA can reverse neurotoxic lipid profiles to a more neuro-regenerative composition. Specifically, this supplementation decreases LCSFA LPC and LPE levels while increasing DHA-containing PC, LPE, and LPC. As DHA supplementation is known to be safe for patients with T2DM, further studies are needed to define its potential role in the treatment of pDN.

### 4.2. Implications of Associated Markers of Neuronal Protection After DHA-Rich Dietary Intervention

Current pharmacotherapies fail to target the underlying nerve damage associated with pDN and expose the user to significant side effects [52]. This lack of effective therapies for pDN has prompted investigators to explore targeting central mechanisms of neuronal homeostasis to prevent/reverse the development of pDN. An emerging target is autophagy, an established pathway of critical importance for neuronal health [40,41,53,54]. Specifically, autophagy is an attractive target since it plays a critical role in regulating neuroinflammation and neuronal survival. Animal studies have recently demonstrated that upregulated autophagic activity can directly alleviate neuropathic pain [55,56].

Metabolic states such as T2DM are often marked by lipid substrate overload. This metabolic imbalance is significant, as excessive lipid accumulation is linked to reduced autophagic turnover [57,58]. Our previous research in preclinical models demonstrated that DHA protects SFA overload, with autophagy playing a central role in DHA-mediated neuronal protection from lipotoxicity [17,29]. In the current study, we observed that participants with moderate-to-high pain had significantly increased serum ATG5 levels after three months of DHA-rich supplementation. These findings reinforce the potential of targeting central mechanisms of neuronal homeostasis, such as autophagy, as an effective strategy to improve clinical outcomes in patients with pDN.

In our intervention, we focused on increasing omega-3 PUFAs in our participants’ diets; therefore, we measured the levels of FABP5, a fatty acid-binding protein involved in neuronal protection and fatty acid transport. Previously, our group and others showed that FABP5 protects neuronal models from lipotoxicity and increases after omega-3 dietary supplementation, and deficiency in FABP5 is associated with neuronal fragility [16,27,29,46,59,60]. In the present study, FABP5 serum levels increased in the participants after the dietary DHA-rich supplementation. This finding, along with our previous reports [31], supports the role of FABP5 in protecting the nervous system from lipotoxicity, oxidative stress, and inflammatory damage, pathways associated with neurodegeneration and pain [27,29,32]. As FABP5 and autophagy processes appear to be central to DHA action, further study is needed on the potential FABP5 involvement in autophagic activity during DHA regulation.

### 4.3. Study Limitations and Strengths

We previously addressed the limitations of this study in detail [31,32]. In summary, the analysis lacks a traditional control group, and participants engaged in an interactive, supportive environment, which may have introduced a Hawthorne or placebo effect on the reported SF-MPQ pain scores.

Confounding factors often complicate the evaluation of outcomes in community-based interventions, particularly when reliable quantitative measures are limited or the inclusion of a control group conflicts with the study’s objectives [61]. To address these challenges, we employed a paired analysis approach, using each participant as their own control and assessing changes relative to baseline values [62]. A key strength of this study lies in its use of lipidomic profiling, which offered detailed insights into the impact of DHA-rich supplementation. The untargeted lipidomic analysis identified significant changes in several lipids associated with DHA metabolism, further highlighting the intervention’s biochemical effects.

## 5. Conclusions

In conclusion, this study highlights the potential of docosahexaenoic acid (DHA)-rich supplementation to modify circulating lipid profiles and reduce neurotoxic lipid mediators associated with painful diabetic neuropathy (pDN) in individuals with type 2 diabetes mellitus (T2DM). The intervention not only significantly decreased neurotoxic lipids but also increased levels of neuroprotective markers, including ATG5 and FABP5, while reducing MLKL, a biomarker of necrosis. These findings align with the established roles of omega-3 polyunsaturated fatty acids in mitigating neuroinflammatory processes and alleviating symptoms of neuropathic pain. This research provides valuable insights into the mechanisms underlying DHA’s therapeutic effects and underscores the potential of lipid-targeted interventions as complementary strategies for managing chronic neuroinflammatory diseases and neuropathic pain.

## Figures and Tables

**Table 1:** Untargeted fatty acid compound identification and statistical comparison.

Total biochemicals identified	283
Total biochemicals *p* ≤ 0.05, adjusted *p*-value (FDR) cutoff, matched paired *t*-test	81
Biochemicals (↑|↓)	**22**|**59**

**Table 2:** Significantly different fatty acids post-DHA-rich supplementation.

Group	Lipid Class	Name	*p*-Value	FDR
Neutral Lipids	Cholesteryl ester (CE)	CE [FA22:6]	1.8 × 10^−12^	7.1 × 10^−11^
		CE [FA22:4]	2.7 × 10^−8^	4.3 × 10^−7^
		CE [FA20:4]	1.3 × 10^−5^	0.0001
		CE [FA20:3]	0.0004	0.002
		CE [FA20:5]	0.0009	0.005
		CE [FA20:0]	0.004	0.016
	Monoacylglycerol (MAG)	MAG [FA18:0]	2.1 × 10^−8^	3.5 × 10^−7^
		MAG [FA16:0]	3.9 × 10^−7^	4.7911 × 10^−6^
		MAG [FA18:1]	0.0006	0.003
	Triacylglycerol (TAG)	TAG [FA22:6]	3.5 × 10^−11^	8.5 × 10^−10^
		TAG [FA22:4]	0.0004	0.002
		TAG [FA20:2]	0.0015	0.0075
		TAG [FA20:3]	0.002	0.01
		TAG [FA20:5]	0.005	0.02
	Diacylglycerol (DAG)	DAG [FA22:6]	2.1 × 10^−5^	0.00019
		DAG [FA20:3]	0.0001	0.00095
		DAG [FA20:2]	0.0002	0.001
		DAG [FA16:0]	0.005	0.02
		DAG [FA20:5]	0.006	0.02
		DAG [FA22:4]	0.009	0.03
		DAG [FA20:4]	0.01	0.047
	Free fatty acid (FFA)	FFA [FA22:6]	1.8 × 10^−12^	7.1 × 10 ^−11^
		FFA [FA20:4]	5.8 × 10^−5^	0.0004
		FFA [FA20:3]	0.0015	0.0077
		FFA [FA16:0]	0.009	0.03
Phospholipids	Phosphatidylcholine (PC)	PC [FA22:6]	1.8 × 10^−12^	7.1 × 10^−11^
		PC [FA22:4]	1.8 × 10^−11^	4.9 × 10^−10^
		PC [FA20:4]	3.5 × 10^−6^	3.5 × 10^−5^
		PC [FA20:5]	3.8 × 10^−5^	0.0003
		PC [FA22:5]	6.3 × 10^−5^	0.0004
		PC [FA14:1]	0.002	0.01
		PC [FA20:0]	0.0046	0.019
		PC [FA17:0]	0.009	0.03
		PC [FA20:3]	0.01	0.046
	Phosphatidylethanolamine (PE)	PE [FA22:5]	3.6 × 10^−12^	1.2 × 10^−10^
		PE [FA22:6]	9.1 × 10^−12^	2.7 × 10^−10^
		PE [FA22:4]	2.0 × 10^−10^	4.2 × 10^−9^
		PE [FA20:5]	3.5 × 10^−6^	3.5 × 10^−5^
		PE [FA20:4]	0.00047	0.0028
		PE [FA20:3]	0.0089	0.03
	Phosphatidylinositol (PI)	PI [FA22:6]	1.8 × 10^−12^	7.1 × 10^−11^
		PI [FA22:5]	0.0038	0.016
		PI [FA18:1]	0.0075	0.028
	Lysophosphatidylcholine (LPC)	LPC [FA22:6]	1.8 × 10^−12^	7.1 × 10^−11^
		LPC [FA20:4]	3.7 × 10^−9^	6.7 × 10^−8^
		LPC [FA16:0]	7.3 × 10^−8^	9.9 × 10^−7^
		LPC [FA22:5]	1.6 × 10^−7^	2.1 × 10^−6^
		LPC [FA16:1]	5.8 × 10^−7^	6.9 × 10^−6^
		LPC [FA22:4]	1.7 × 10^−6^	1.8 × 10^−5^
		LPC [FA20:2]	3.1 × 10^−5^	0.0003
		LPC [FA17:0]	5.1 × 10^−5^	0.0004
		LPC [FA20:3]	5.1 × 10^−5^	0.0004
		LPC [FA18:0]	6.3 × 10^−5^	0.0004
		LPC [FA20:5]	0.0005	0.003
		LPC [FA20:1]	0.0006	0.003
		LPC [FA18:1]	0.0006	0.003
		LPC [FA15:0]	0.001	0.009
		LPC [FA14:0]	0.01	0.04
	Lysophosphatidylethanolamine (LPE)	LPE [FA22:6]	1.8 × 10^−12^	7.1 × 10^−11^
		LPE [FA22:5]	4.6 × 10^−10^	8.9 × 10^−9^
		LPE [FA18:0]	51 × 10^−8^	7.65 × 10^−7^
		LPE [FA20:4]	6.46 × 10^−8^	9.2 × 10^−7^
		LPE [FA20:5]	3.2 × 10^−5^	0.00026
		LPE [FA22:4]	5.6 × 10^−5^	0.0004
		LPE [FA20:3]	0.0004	0.002
		LPE [FA16:1]	0.00056	0.003
Sphingolipids	Sphingomyelin (SM)	SM [FA26:0]	0.01	0.04
	Ceramide (CER)	CER [FA22:0]	0.0035	0.015
		CER [FA20:0]	0.0055	0.02
		CER [FA16:0]	0.01	0.044
	Dihydroceramide (DCER)	DCER [FA26:0]	8.8 × 10^−6^	8.6 × 10^−5^
		DCER [FA24:0]	0.002	0.01
		DCER [FA22:0]	0.007	0.026
Total	N/A	Total [FA22:6]	1.8 × 10^−12^	7.1 × 10^−11^
		Total [FA22:4]	1.0 × 10^−10^	2.3 × 10^−9^
		Total [FA20:4]	1.5 × 10^−6^	1.7 × 10^−5^
		Total [FA20:5]	0.0002059	0.001366
		Total [FA20:3]	0.00075	0.004
		Total [FA26:0]	0.0015	0.0075
		Total [FA20:2]	0.0055	0.02
		Total [FA22:5]	0.0055	0.02

Significant fatty acid increases and decreases are highlighted in red and blue.

## Data Availability

Data supporting reported results can be found in a dataset generated during the study. Additional information is available upon request.
